# Strategies for whole-exome sequencing analysis in a case series study of familial male infertility

**DOI:** 10.18502/ijrm.v13i5.7158

**Published:** 2020-05-31

**Authors:** Masomeh Askari, Dor Mohammad Kordi Tamandani, Navid Almadani, Mehdi Totonch

**Affiliations:** ^1^Department of Biology, Sistan and Baluchestan University, Zahedan, Iran.; ^2^Department of Genetics, Reproductive Biomedicine Research Center, Royan Institute for Reproductive Biomedicine, ACECR, Tehran, Iran.; ^3^Department of Stem Cells and Developmental Biology, Cell Science Research Center, Royan Institute for Stem Cell Biology and Technology, ACECR, Tehran, Iran.

**Keywords:** Male infertility, Whole-exome sequencing, GATK, SAMtools.

## Abstract

**Background:**

Infertility is one of the common health issues around the world. The prevalence of male factor infertility among infertile couples is approximately 30%-35%, of which genetic factors account for 15%. The family-based whole-exome sequencing (WES) approach can accurately detect novel variants. However, selecting an appropriate sample for data generation using WES has proven to be challenging in familial male infertility studies. The aim of this study was to identify types of pathogenic male infertility in cases of familial asthenozoospermia.

**Case:**

Two families with multiple cases were recruited for the purpose of WES. The study population included two affected cases in pedigree I and three affected cases in pedigree II. Two different variant callers (SAMtools and GATK) with a single-sample calling strategy (SSCS) and a multiple-sample calling strategy (MSCS), were applied to identify variant sites.

**Conclusion:**

In this study, we represented the results for variant prioritization of WES data without sequencing fertile siblings in the same pedigree by applying two different pipelines (homozygosity and linkage-based strategy). Using the aforementioned strategies, we prioritized annotated variants and generated a logical shortlist of private variants for each pedigree.

## 1. Introduction

Infertility is an important social health condition, and is defined as the inability to conceive naturally after at least one year of unprotected intercourse. Approximately 15% couples suffer from infertility and 30%-35% of those cases relate to male factor infertility (1). Numerous factors (ranging from lifestyle to heredity) may affect semen parameters and male infertility (2). Approximately 15% of male infertility cases are attributable to genetic disorders (3). The majority of mechanisms involved in disease heritability are associated with chromosomal abnormalities and genetic variations (4). According to OMIM, autosomal or sex chromosome has more than 60 genes, leading to male infertility. CFTR mutation has been implicated in 60%-90% of cases involving infertile men, with congenital bilateral absence of the vas deferens or epididymal obstruction. Mutations in X-linked genes, such as *USP26* and *SOX3*, have been reported in cases of severe spermatogenesis impairment (5, 6).

Approximately 30%-40% infertility cases in men with unknown etiology may be associated with genetic factors (6). The high proportion of idiopathic infertility in males is attributed to the limitations of traditional gene identification techniques, such as the Sanger sequencing. The most notable disadvantages of these approaches include length of time, low throughput data, and a broad region for candidate genes. Most of these limitations have been addressed by next-generation sequencing (NGS) (7).

The NGS approach, or whole-exome sequencing (WES), is a powerful and unbiased tool for identifying genetic variation by capturing genome coding regions (8). WES covers a region of approximately 1%-1.5% of the human genome, where approximately 85% of causative mutations are located. Overlap-, de novo-, extreme phenotypes, and familial-based are the four main strategies for WES analysis. Of these, family-based WES is an efficient method of identifying potential causal variants (9). This approach led to the identification of causal variants that traditional methods had failed to detect in several pedigrees (10). The WES specialist can generate large amounts of data and, consequently, be of benefit to clinics. However, excluding the called variations across the whole-exome remains an issue, and it is not reasonable to verify all types of candidates via the Sanger sequencing (11, 12). Indeed, prioritizing the best candidate pathogenic variants is the main challenge. Many previous studies have focused on the family-based strategy to increase the efficiency of discovering candidate genes and reducing the number of private variants (13).

The purpose of this study was to illustrate the basic familial framework for the purpose of identifying genes that relate to male infertility. To this end, we studied seven cases in two families. In family I, we sequenced two affected members and their parents to filter out inherited variants. In family II, we focused on the most distantly related family members to reduce shared benign variations. The advantage of this approach, by sequencing non-affected siblings, is that not many private variations were missed.

## 2. Case

### Human subjects and DNA samples

**Figure 1:**
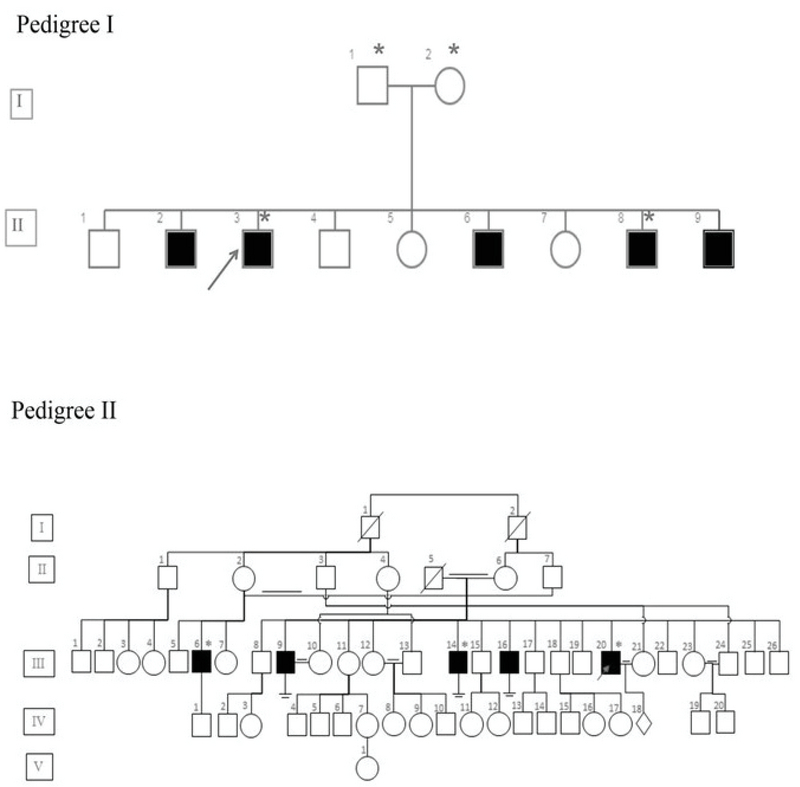
Pedigree of families with ASZ. Pedigree I and pedigree II. The black and white squares represent infertile and fertile men, respectively. The proband is indicated by a red arrow. Candidate cases that underwent WES are indicated by a red asterisk.

In this case series study, we examined seven cases from two unrelated families (Figure 1). DNA was extracted from the peripheral blood leukocyte using salting out methods.. The quality of the DNA was checked using an agarose gel and NanoDrop analysis.

### Whole-exome sequencing platform

The library of all seven samples was prepared using the SureSelectXT Library Preparation Kit (Agilent Technologies, Santa Clara, CA, USA). As a next step, cluster generation, paired-end sequencing was applied on the IlluminaHiSeq 2000 platform using TruSeqv3. The binary base call (BCL) was converted to FASTQ using the Illumina bcl2fastq package.

### Quality evaluation of the raw data

The preprocessing and generation of raw reads (FASTQ) involved 3´ end adaptor clipping, primer removal, and the trimming of poor base sequence quality. The sequenced data were assessed using the FastQC tool (http://www.bioinformatics.babraham.ac.uk/projects/fastqc/) to analyze Phred score distribution, together with reads, GC content distribution, read length distribution, and sequence duplication level. The adaptor was removed using Trimomatic (version 1.04.636; www.github.com/ExpressionAnalysis/ea-utils/blob/wiki/FastqMcf.md).

### Alignment and duplicated PCR removed

Following the assessment of quality control, the raw reads were aligned to an established human reference genome (version hg19UCSC indexed in the FASTA format (14). Millions of reference genome-scale short reads have been globally streamlined using the Burrows-Wheeler Aligner (BWA) with MEM algorithm alignment in SAM format (15). The default parameters in the software were based on the Burrows-Wheeler conversion. The aligned reads were converted to a BAM file using SAMtools software (16), which was able to clean up and flag read-pairing information. The BAM file was then sorted by genomic location and indexed by SAMtools to save space and help the subsequent process. The Picard tool was combined with bamtools to filter out the mismatching and inappropriate reads for the purpose of assembled genomic data. The Picardtool identified duplicates from the PCR library. Data distribution and reads coverage (alignment statistics) were then evaluated with the CalculateHsMetrics package. Base quality was recalibrated, using GATK covariance recalibration (version 2.8; Broad Institute, Cambridge, MA, USA; www.broadinstitute.org/gatk/).

### Variant calling and annotation

Variant identification is the key stage in NGS data analysis. In this step, two programs, GATK and SAMtools, were applied to create a VCF file containing all the sites with potential variants (16). VCF files were filtered based on two criteria: depth of coverage and Fred score quality (DP > 9 -'QUAL > 25). After calling variants list,, the ANNOVAR software tool was used to annotate screened variations and connect the three annotation modes according to the type of gene, region, and filter (17).

### Variant filtering

The number of candidate variants was reduced through four steps. The first step was to filter the variants out of the coding or splicing region. The second step was to prioritize the variants with relatively low minor allele frequency (MAF <1%), or novel variants. The third step was to exclude synonymous variants and retain splice, nonsense, Indel, and nonsynonymous mutations. To reduce the number of candidate genes, we focused on the identification of genes related to two strategies (homozygosity and linkage-based). Family I underwent homozygosity strategy because the DNA samples of both parents were available. Family II was selected for linkage-based strategy because DNA samples from all affected cases were available. Five infertile men in this family were diagnosed with asthenozoospermia (ASZ); the DNA samples of two brothers and one of the most distant affected relatives were sequenced to identify the shared variations. In addition, the homozygosity region for each family was obtained using exome data. A homozygous variant in the homozygous region was prioritized.

### Variant analysis 

Specific variants were assessed for evolutionary conservation using the GREP and PhastCons tools. Variants with a GREP score of >2.0 and a PhastCons score of >0.3 were considered a conserved variant. The prediction of candidate variants that affected protein or phenotype function was performed using five tools: Mutation Taster, PolyPhen, Sift, CADD, and Proven (18). The variants predicted to be disease-causing by more than two of these tools were further analyzed. A deleterious effect of variants on protein structure was evaluated by the online web service HOPE. Pathogenic variation is able to disrupt intron-exon splice sites, exonic splicing enhancers (ESE) and exonic splicing silencers (ESS), which can affect gene expression and cause genetic diseases by aberrant pre-mRNA splicing. The annotation of intronic and exonic mutations, leading to splicing defects, was performed using the ESEfinder and Human Splicing Finder web resource tools (19, 20).

### Ethical consideration

As a first step, written informed consent for genetic studies was obtained from subjects. This case series study was conducted in accordance with the protocols approved by the Institutional Review Board of the Royan Institute Research Center and the Royan Ethics Committee, Tehran, Iran.

### Bioinformatics tools

• FastQC tool

• Trimomatic

• Burrows-Wheeler Aligner

• SAMtools

• BamTools

• Picard tool

• CalculateHsMetrics package

• GATK

• ANNOVAR

## 3. Results

### WES Run Statistics and alignment results

Approximately 2.5 gigabytes of exome sequence were obtained for each of the seven samples. All sequence sets yielded a comparable total number of bases, reads, GC (%), Q20 (%), and Q30 (%). Table I shows the raw stats data of each sample. FastQC was performed to check the quality of the raw data, focusing on base quality score, GC content, N content, and sequence duplication level. Mapped results, previously generated by BWA, were computed to create the number of total reads, paired reads, properly-paired reads, duplicate reads, duplication rate percentage (number of duplicates/total reads), number of reads mapped/aligned, and unique reads mapped (Table II).

### Variant detection 

**Figure 2:**
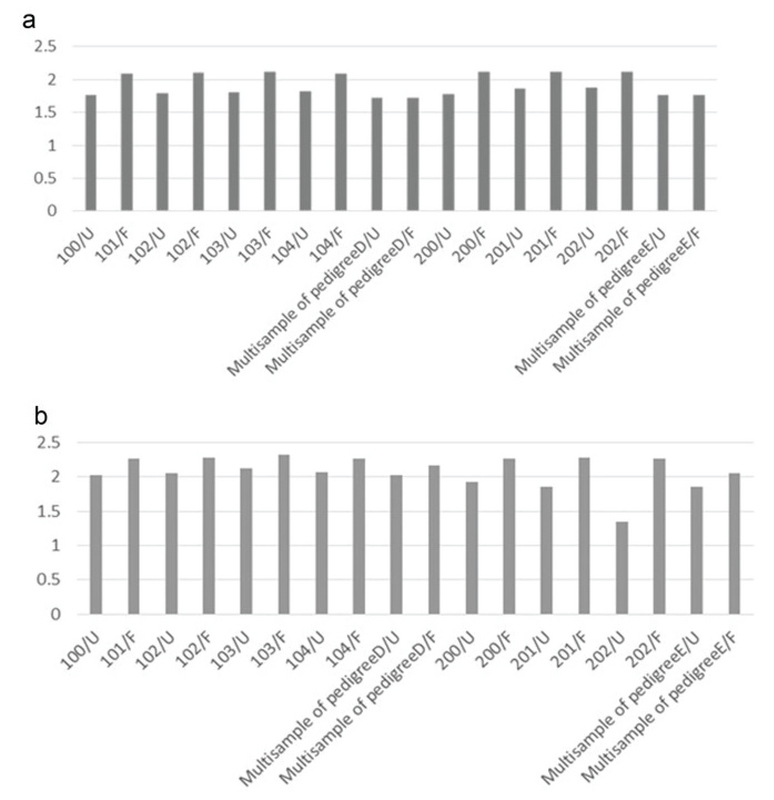
Comparison of Ts/Tv between filtered and unfiltered variants. (a) Ts/Tv ratio of unfiltered (U) and filtered (F) variants called by SAMtools; (b) Ts/Tv ratio of unfiltered (U) and filtered (F) variants called by GATK.

**Figure 3:**
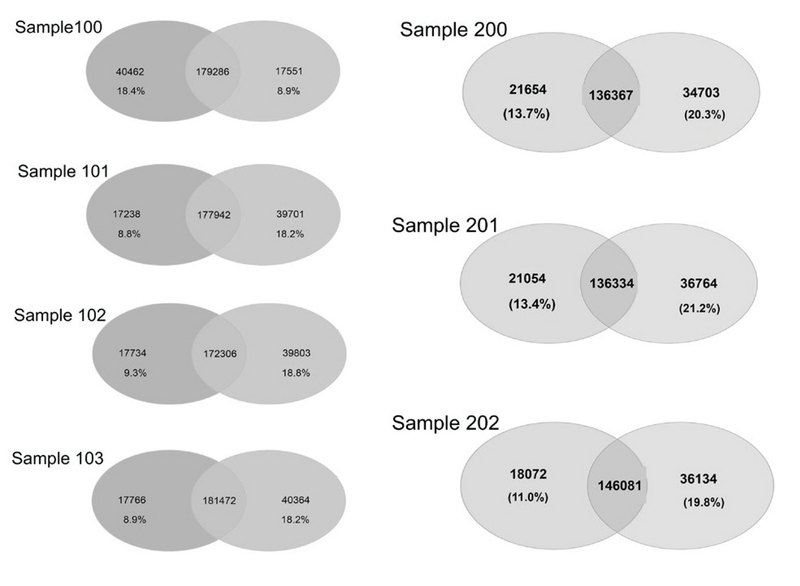
The intersection of variants identified by the two-caller strategy**.** The Venn diagram depicts the number of variants in the seven cases, identified by GATK and SAMtools.

Variants were called using two different tools, GATK (version 2.6) and SAMtools (version 0.1.18), which are the most widely used, and the Unified Genotyper algorithm was applied. SSCS and MSCS were separately performed for family I and family II. The number of SNPs and Indels was determined by different callers, and we evaluated the effects of SSCS and MSCS on the variant for each family (Table III). Through SAMtool, SSCS increased the number of raw SNPs by 1.6% and 1.1% in family I and family II, respectively. In contrast, MSCS increased the number of raw SNPs called by 1.2% with GATK in both pedigrees. Variants were filtered based on the depth of coverage and quality. In SSCS and MSCS, variant filtering removed 15% and 21% of raw variants called by SAMtools, respectively, whereas 16% and 9.2% of those called by GATK were filtered. These results reflect the importance of filtering in MSCS by SAMtools. We estimated the Ts/Tv ratio of SNP sets using two SSCS and MSCS pipelines. As shown in Figure 2a, in SSCS with SAMtools, the raw SNP sets had a Ts/Tv ratio of between 1.72 and 1.88, while the Ts/Tv ratio increased in the filtered data sets. The Ts/Tv ratio was constant in both unfiltered and filtered MSCS data sets with SAMtools. The Ts/Tv ratio of the raw SNP sets was 1.34-2.07 when the GATK was used, and increased in both SSCS and MSCS with GATK (Figure 2b). The results demonstrate that 83%-91% of SNVs are called by both GATK and SAMtools (Figure 3).

### Variant filtering

In this stage, the called variants were filtered in four stages. In the first stage, we excluded approximately two-thirds of variants, which were located in the intronic and intergenic regions. Less than 50% of variants were likely to be pathogenic. Of these, new and rare variants with MAF <1% required further analysis (Table IV). As the affected cases in family II were from a consanguineous marriage, we focused on homozygote variants. The number of private variants in pedigree II is shown in Table V. Approximately 0.05% (SAMtools) and 0.03% (GATK) of the called variations remained after initial filtering in family I (Table VI).

**Table 1:** NGS run stats for seven samples


**Sample ID**	**Total read bases (bp)**	**Total reads**	**GC (%)**	**AT (%)**	**Q20 (%)**	**Q30 (%)**
**100**	10,374,885,048	68,707,848	52.49	47.51	96.84	94.94
**101**	9,551,889,446	63,257,546	52.31	47.69	96.79	94.86
**102**	8,128,247,252	53,829,452	52.79	47.21	95.77	93.28
**103**	10,019,530,104	66,354,504	51.95	48.05	96.79	94.86
**200**	9,915,418,624	65,665,024	52.94	47.06	96.4	94.29
**201**	10,637,953,322	70,450,022	53.05	46.95	96.43	94.33
**202**	9,679,497,432	64,102,632	53.05	46.95	96.59	94.58
Total read bases: Total number of bases sequenced. Total reads: Total number of reads. For Illumina paired-end sequencing, this value refers to the sum of read 1 and read 2. GC (%): GC content. AT (%): AT content. Q20 (%): Ratio of bases that have a Phred quality score in excess of 20. Q30 (%): Ratio of bases that have a Phred quality score in excess of 30						

**Table 2:** Alignment statistics for seven data aligned with BWA MEM


**Samples **	**Paired reads in sequencing**	**Properly paired**	**Read 1**	**Read 2**	**Mapped reads**	**Unmapped reads**	**Read-pair optical duplicates**	**Duplication percentage **
**100**	68,707,848	67,621,688	34,353,924	34,353,924	68,767,520	65,069	424,113	0.150270
**101**	63,257,546	61,244,026	31,628,773	31,628,773	63,328,202	61,270	401,670	0.151691
**102**	53,829,452	52,945,158	26,914,726	26,914,726	53,969,040	49,300	315,834	0.141146
**103**	66,354,504	64,700,696	33,177,252	33,177,252	66,417,218	64,207	445,562	0.159041
**200**	65,665,024	63,758,102	32,832,512	32,832,512	65,809,832	249,151	19,714	0.144086
**201**	70,450,022	68,075,954	35,225,011	35,225,011	70,636,251	249,453	21,555	0.274010
**202**	64,102,632	63,080,570	32,051,316	32,051,316	64,296,060	222,177	27,934	0.057078

**Table 3:** Number of SNPs and Indels called by SAMtools and GATK


**Number of variants **	**Unfiltered SNP**		**Filtered SNP**		**Unfiltered indel**		**Filtered indel**	
	**SAMtools**	**GATK**	**SAMtools**	**GATK**	**SAMtools**	**GATK**	**SAMtools**	**GATK**
**100**	1,020,180	492,537	183,178	103,790	91,216	53,722	22,058	11,750
**101**	1,073,008	518,360	181,294	102,548	93,768	56,052	21,935	11,474
**102**	930,610	456,647	179,665	100,100	82,297	48,705	21,764	11,242
**103**	1,066,604	458,137	177,006	95,249	86,249	46,024	20,276	9,479
**Multiple samples of pedigree I**	2,557,705	2,396,362	169,315	260,117	210,415	153,486	37,415	32,228
**200**	1,290,000	870,536	140,444	82,796	85,664	76,129	17,204	9,612
**201**	1,624,699	190,435	141,757	79,148	110,845	84,310	17,363	8,887
**202**	1,631,100	723,156	149,358	104,198	111,435	65,608	18,524	12,024
**Multiple samples of pedigree II**	4,013,542	2,265,075	190,882	137,137	245,998	194,616	22,765	24,739
SNP: Single nucleotide polymorphism, Indel: Insertion and deletion								

**Table 4:** Distribution of variant type called by GATK and SAMtools from seven samples


**Individual **	**100**	**101**	**102**	**103**	**200**	**201**	**202**
**Variants called with GATK**	114,371	111,669	10,5012	115,909	92,631	88,211	39,347
**Exonic, exonic/splicing Splicing**	41,505	41,008	40,454	41,884	34,597	33,708	27,961
**Nonsynonymous Indel**	29,898	29,233	28,439	29,962	23,504	22,671	9,584
**Rare and novel **	2,848	2,831	2,607	2,776	2,106	2,215	2,848
**Variants called with SAMtools**	204,543	202,734	198,344	206,533	158,404	159,939	168,858
**Exonic, exonic/splicing Splicing**	55,066	55,106	54,821	55,633	45,814	45,683	47,165
**Nonsynonymous Indel**	43,235	43,113	42,529	43,530	34,330	34,233	35,637
**Rare and novel **	9,679	9,482	8,956	9,545	7,925	6,150	7,340

**Table 5:** Private variants of pedigree II called with SAMtools and GATK


**Individual**	**100**		**101**		**102**		**Common in three affected**	
	**SAMtools**	**GATK**	**SAMtools**	**GATK**	**SAMtools**	**GATK**	**SAM tools**	**GATK**
**Nonsynonymous SNV**	35	34	35	51	42	32	5	5
**Frame shift**	84	1	84	1	84	0	65	0
**Indel**	255	3	255	5	264	5	138	0
**Stopgain**	5	0	5	0	5	0	2	0
**Stoploss**	1	1	1	0	1	1	0	0
**5´UTR**	178	10	178	4	213	7	94	0
**3´UTR**	263	8	263	12	276	4	147	2
**Exonic**	329	37	329	60	347	37	140	5
**Exonic, splicing**	47	1	47	0	47	1	7	0

**Table 6:** Private variants of pedigree I called with SAMtools and GATK


**Individual**	**200**		**201**		**202**		**203**		**Case /control**	
	**SAMtools**	**GATK**	**SAMtools**	**GATK**	**SAMtools**	**GATK**	**SAMtools**	**GATK**	**SAMtools**	**GATK**
**Nonsynonymous SNV**	770	698	792	675	820	751	748	663	85	56
**Frame shift**	160	23	148	25	162	24	167	23	23	4
**Indel**	754	60	779	67	726	61	781	54	224	12
**Stopgain**	28	6	33	11	35	12	28	10	8	1
**Stoploss**	33	3	3	1	2	0	7	1	5	0
**5´UTR**	669	84	712	98	603	92	647	88	156	21
**3´UTR**	1001	177	1046	192	934	166	1015	195	322	35
**Exonic**	1718	794	1744	802	1712	854	1667	795	225	75
**Exonic, splicing**	94	10	96	9	93	10	89	13	14	2

## 4. Discussion

NGS-based technology is a powerful tool for identifying the genetic basis of the human phenotype. As one of the leading molecular techniques in the field of reproductive medicine, WES has a major impact on our understanding of the genetic causes of male infertility. WES generates a large amount of genetic data; however, the processing of WES data can be complex (11). As a standard pipeline, BWA aligns the sequencing reads against the reference genome and variant callers detect the SNVs and Indels (7). In this study, SAMtools and GATK were used as variant callers with SSCS and MSCS. Both unfiltered and filtered variants indicated that SAMtools called more SNVs than GATK did owing to its lower internal filtering criteria. Our results demonstrated that filtered and called Indel variants with SAMtools decreased compared with GATK. In parallel, other studies confirmed the high potential of GATK in the identification of true Indel variants (21). In addition, the number of called raw SNVs increased in SSCS with SAMtools and in MSCS with GATK, in both families. The results showed that SAMtools and GATK are capable of calling more raw SNVs in SSCS and MSCS, respectively, in both analyzed pedigrees. Therefore, we suggest that GATK and SAMtools are appropriate callers in MSCS and SSCS, respectively. These results, along with other reports that illustrate the role of GATK in MSCS, such as those by Liu *et al.*, demonstrate that many variants have been lost by SAMtools in MSCS (21). Cornish and Guda compared 30 different pipelines and found that Novoalign plus the GATK Unified Genotyper showed the highest sensitivity with a low number of false positives (22).

Variants were annotated by ANNOVAR, and variants outside the coding regions, as well as those of synonymous coding, were filtered out. Subsequently, known variants with a frequency greater than 1% in ExAC, dbSNP, and 1,000 Genomes were excluded, to reduce the number of potential disease-causing variants (23-25). Homozygosity mapping and linkage approaches prioritized private variants for further analysis based on the two family-based filtering strategies. As shown in Tables V and VI, SAMtools called more private variants that were either missed by GATK or flagged as low-quality.

We have shown that WES is a powerful, efficient, and cost-effective technique that significantly reduces the number of candidate genes in a small number of infertility cases in families with multiple affected individuals. Our research demonstrates that sequencing a small number of samples, while using appropriate filters against public SNVs and in-house databases, is a sufficient approach to detecting private variants. The genomic study of familial male infertility is limited in several respects: the first challenge is the identification of families that contain more than two infertile men who are willing to participate in the study. The selection of appropriate samples for sequencing is also very important. There is a level of uncertainty when detecting genuine fertile men as a result of assisted reproductive technologies, age of incidence, and cultural barriers. Therefore, great care should be taken in the selection of fertile samples.

## 5. Conclusion

This study demonstrates two strategies for the WES analysis of familial male infertility cases to suggest a convenient approach to identify potentially functional variants. This, in turn, may further our understanding of the underlying mechanisms behind male infertility.

##  Conflict of Interest

The authors declare that they have no conflicts of interest.
